# Block Teeth

**Published:** 1861-04

**Authors:** 


### Selections.

---

Block Teeth.—Remarks, *by J. H. McQuillen, D. D. S.* —The valuable method of making block teeth described below *was presented* to the profession, about seven years ago, through the columns of the *Dental News Letter*, (July, 1854.) As the plan, however, does not appear to have attracted that attention which it justly merits, we have deemed it advisable, on account of the great practical value of the communication, to republish it.

There is nothing in mechanical dentistry more difficult to *excel in*, or even to attain to a respectable degree of perfection, than the carving of teeth, and a tacit acknowledgment of the correctness of this assertion is made by the vast majority of practitioners when they employ others to do such work for them. The following plan, however, ought to enable those who possess application and a moderate degree of manipulative skill, to produce work satisfactory alike to themselves and patients.

The late and lamented Dr. Walker, Demonstrator of Mechanical Dentistry in the Pennsylvania College of Dental Surgery, who had a great deal of experience with this method, and regarded it with much favor, produced some of the most natural specimens of artificial teeth in this way that we have ever seen.

It has been stated above that the inventor *presented* his plan to the profession; and we would further remark that, when taking into consideration the valuable character of the present thus voluntarily made, the act is not only worthy of commendation by those who may be benefited by it, but also of imitation by those who, year after year, secure patents for the most useless and nonsensical inventions, which they afterward attempt to foist on the profession.

We take p'easure in stating that the course pursued by Dr. Calvert in this matter was appreciated by his fellow-practitioners in Philadelphia; for, without any solicitation on his part, a committee was appointed by the Pennsylvania Association of Surgeon Dentists to inquire into the merits of the method, and the committee not only submitted a favorable report, but also recommended that a gold medal should be awarded to the inventor, which was accordingly done.

"*A New Method of Making Block Teeth.* By WILLIAM CALVERT, D. D. S.—Permit me, through the medium of your valuable columns, to offer for the consideration of the profession a few brief remarks upon the subject of *block making*, or the manufacture of porcelain block teeth.

"It is not my intention, nor is it my desire, here to treat of the various methods that have from time to time been practiced by those engaged in the manufacture of block teeth. Nor is it my purpose to discuss the *relative merits* of the different methods, or of any one in particular, but my aim shall be to attempt a description of a process of my own that I adopted some time ago, and which I have most successfully practiced.

"The first preparatory step to be taken, after having correct articulating models, is to select single teeth so defined as may either suit the taste of the operator or the peculiarity of the case, and supposing the case to be an upper denture, it will be necessary to have *two* front and two lateral incisors, two canine or cuspids, two bicuspids, (or if more convenient, the cuspids) and four molars, all of which should be sufficiently large to compensate for shrinkage, in the material of which the teeth are to be composed.

"The plate upon which the blocks are to be made, and to which they are to be subsequently fitted, being upon its corresponding model, a rim of wax may be placed upon it, and the teeth arranged upon the wax, articulating with the antagonizing model, allowing sufficient in the length of the teeth for shrinkage. Beginning with the front incisors, the teeth should be set to the wax (as above) as far back on each side as the first bicuspids, inclusive; then leaving a space equal to the width of *half a tooth*, the arch may be completed by the addition of the molars, two on each side. The teeth having been thus arranged upon the wax, with reference to regularity or irregularity, height, etc., the desired outline of gum may be filled up with wax.

"Special care is requisite in so trimming the wax where joints are contemplated, that no subsequent alteration will be needed during the further manipulations.

"It will be necessary, previous to making the models, to make some provision for replacing them, after they have been once removed, so that they shall occupy the same position as they did previous to their first removal. For this, it will be only necessary to make some conical holes in the face of the cast, say two on each side, between the center and the first bicuspid teeth, and two opposite the molar teeth of each side. These holes need not be more than about a quarter of an inch deep, and should be but a short distance below the edge or line of the plate. The face of the cast, including said holes, should now be varnished, when the case is ready for making the moulds.

"The first mould to be made should be that including the four incisors, two canine, and two first bicuspids, eight teeth in all. This may be done by simply oiling the face of the teeth, outline of gum, and plaster cast, and pouring plaster of Paris of a proper consistency over the surface of the same, allowing it to fall slightly over the cutting edges, so as to form a more perfect mould. This mould should be divided in the center, making two sections, which can be done by cutting through the plaster while in the state of hardening; or, what is perhaps better, before applying the plaster, make an incision in the wax outline of gum, in which place a thin slip of sheet-lead, letting it extend a little above the cutting edges of the teeth, and as far down the face of the cast as is desired to extend the mould. When hard, remove from the cast and teeth, and we have the untrimmed mould for said eight teeth. Previous to making the moulds for the back teeth, it is necessary to remove the *first* bicuspids, or the cuspids representing them, from the position they occupied in making the mould just described, and placing them beside the first molars so as to represent the *second* bicuspids. Care is to be taken in removing and replacing them, so that the original form of the wax be preserved, otherwise the end thereby intended to be secured will be defeated, and the joints at these points will be irregular and unsightly.

"For the purpose of rendering clear a point necessarily left somewhat obscure in the foregoing description, it may be well here to state that the space of *half a tooth*, left between the first bicuspids and the first molars, is to compensate for shrinkage in the length of the arch, for after the first bicuspids are removed and set adjacent to the first molars, thereby representing the second bicuspids, they occupy the entire vacancy first left and one-half the space formerly occupied by said first bicuspids; hence the extension of the back moulds toward the center is equivalent to the shrinkage of the entire arch.

"As the foregoing is applicable where the case of fourteen teeth is to be divided into four blocks, as is usual in soldering, I would say that when the intention is to make pin holes for riveting, the space of *half a tooth* must be left between the canine and bicuspids, instead of between the bicuspids and molars.

"The moulds for the back teeth may now be made in the same manner as those of the front ones. After the moulds have been made as already described, they should be so trimmed that in the process of moulding the blocks there would be no liability of removing portions of the enamel off the teeth in withdrawing the moulds. The moulds should now be varnished with some spirit varnish, and after it becomes dry are ready for use.

"The moulds being prepared, the next step is the enameling of the teeth in the moulds. The enamels should be moistened with a little clean water, and having previously oiled the section or sections of the mould, the blue or point enamel may be first applied (as stiff as it will work) with a very small spatula made for the purpose. The enamel should be *thin* at the base, and gradually thickening with the concavity of the mould to the cutting edges of the teeth. The yellow or base enamel is next applied *heavy* at the base, and gradually terminating near the point.

"After the enameling has been completed so far as is designed to be moulded at one time, a small quantity of the body about the consistency of a thick paste may be spread over the surface of the moulds and of the enamels, the moulds replaced upon the model, and the body carefully filled in, at first rather soft, but subsequently harder and harder, until the mould is sufficiently full. Then applying the flame of a spirit-lamp for a few minutes with the blow-pipe, the body will be toughened enough to work well, when the moulds may be removed. The teeth may then be separated and trimmed, the blocks divided as desired, the *gum* enamel applied, etc., etc., and so completed.

"The process of enameling and moulding being precisely the same with all the blocks, it needs not that I should go into further detail.

"I have already said, that when the blocks are intended to be riveted upon the plate, the moulds are required to be somewhat different. There is also another difference; that is, the moulding of the pin or rivet holes, which may be done by removing the plate from the model, placing the moulds upon the model, and drilling a small hole upon the prominence of the ridge opposite the center of each tooth, in which insert a piece of wire of a desired size. The enameling, etc., may then be done as before described, and after the body has been hardened sufficiently, the pins may be removed, leaving the holes neatly moulded, perfectly smooth, and straight. The blocks may then be finished at once, before removing from the cast."—*Dental Cosmos.*

